# Imaging of nerve injury in neonatal acute bilirubin encephalopathy using ^1^H-MRS and Glu-CEST techniques

**DOI:** 10.3389/fnins.2023.1110349

**Published:** 2023-03-28

**Authors:** Qihuan Lin, Lanmei Chen, Hongyi Zheng, Hui Tan, Gengbiao Zhang, Wenbin Zheng

**Affiliations:** ^1^Imaging Center, Nanfang Hospital, Southern Medical University, Guangzhou, Guangdong, China; ^2^Department of Radiology, The Second Affiliated Hospital, Shantou University Medical College, Shantou, Guangdong, China

**Keywords:** acute bilirubin encephalopathy, proton magnetic resonance spectroscopy, glutamate chemical exchange saturation transfer, brain, nerve injury

## Abstract

**Objectives:**

To investigate the significance of proton magnetic resonance spectroscopy (^1^H-MRS) and glutamate chemical exchange saturation transfer (Glu-CEST) techniques in assessing the condition and prognosis of acute bilirubin encephalopathy patients and to understand the mechanism of nerve injury in this disease.

**Materials and methods:**

From September 2019 to February 2021, 31 neonates with acute bilirubin encephalopathy and 16 healthy neonates were enrolled in this study. All the quantitative results of ^1^H-MRS, Glu-CEST, and conventional magnetic resonance imaging (MRI) of all neonates were analyzed. The associations between statistically significant indicators of imaging and developmental quotients (DQ) were analyzed.

**Results:**

The 31 cases were assigned to the mild subgroup (*n* = 21) and moderate and severe subgroup (*n* = 10) according to the bilirubin-induced neurologic dysfunction (BIND) scores. The case group had elevated Cho and GABA absolute concentrations compared to the normal control group (all *p* < 0.05). Compared with the normal control group, the absolute concentration of GABA of the moderate and severe subgroup was significantly larger (*p* < 0.05). Compared with the normal control group, the Glu-CEST% values in the left basal ganglia, right thalamus, left frontal cortex and bilateral medial geniculate body of the case group was significantly larger (all *p* < 0.05). The moderate and severe subgroup had higher Glu-CEST% values in the left basal ganglia, right thalamus, and bilateral medial geniculate body than the normal control group (all *p* < 0.05). A negative association was revealed between the DQ scores and the Glu-CEST% values in the left basal ganglia (*r* = −0.888, *p* < 0.05).

**Conclusion:**

The combination of ^1^H-MRS and Glu-CEST techniques can monitor the intracerebral metabolite level of acute bilirubin encephalopathy and evaluate the illness severity.

## Introduction

Neonatal hyperbilirubinemia is one of the most common reasons for neonate medical care visit and admission. Neonatal jaundice is divided into physiological jaundice and pathological jaundice. Physiologic jaundice usually occurs within 2–4 days of birth and serum TSB levels in full-term infants are usually not more than 12.9 mg/dl. Physiologic jaundice resolves within 2 weeks at most. Premature infants with total bilirubin levels below 15 mg/dl may delay complete resolution to 3–4 weeks (Hansen, 2002). However, when jaundice occurs within 24 h after birth, bilirubin concentration is higher than the above value, and jaundice lasts for a long time and recurs, which can be considered as pathological jaundice. Extreme bilirubin concentrations in the early stage might lead to kernicterus which is the most severe complication of neonatal hyperbilirubinemia. Magnetic resonance imaging (MRI) is of great significance for confirming neonatal bilirubin encephalopathy, which is easy, noninvasive, and widely applied in clinical practice.

Neonates with acute bilirubin encephalopathy usually manifest symmetrical hyperintensity on T1WI in the globus pallidus and thalamus at early conventional MRI scan, Wisnowski et al. (2014). The pathological basis for the aberrant signal intensity is presumably due to bilirubin neurotoxicity injuring nerve cell membranes caused by astrocyte response and bilirubin accumulation within neurons (Rodrigues et al., 2002). However, some neonates with hypoxic ischemic encephalopathy (HIE) and normal neonates might also exhibit T1WI hyperintensity in bilateral globus pallidus (Barkovich, 1998; Wisnowski et al., 2014). Meanwhile, the signal intensity was determined by subjective judgment, so false positive was unavoidable. Therefore, in clinical practice, more sequences are needed to assess the condition of patients with acute bilirubin encephalopathy to administer appropriate treatment and reduce sequela occurrence.

Proton magnetic resonance spectroscopy (^1^H-MRS) is a valuable noninvasive technique that monitors brain metabolism *in vivo*. Previous studies regarding bilirubin encephalopathy MRS showed that neonates with bilirubin encephalopathy had higher Cho/Cr ratios versus healthy neonates (Sarı et al., 2015). A study conducted by Oakden WK (Oakden et al., 2005) demonstrated that the case group had higher Tau/Cr ratios versus the normal control group. Wu et al. (2013) reported that the NAA/Cr ratios of basal ganglia increased with increasing peak serum total bilirubi (TSB) levels in the case group.

Glutamate chemical exchange saturation transfer (Glu-CEST) is a new noninvasive *in vivo* imaging method for oriented brain glutamate analysis and has markedly greater sensitivity and higher spatial resolution than the conventional ^1^H-MRS method (Cai et al., 2012). The Glu-CEST technique has been applied in analyzing the healthy human brain and spinal cord. Based on numerous studies at home and abroad, glutamic acid is considered a crucial amino acid in human emotion, learning, memory, and cognitive function, extensively involved in almost all signal-processing functions of the central nervous system. It modulates neuron survival, dendrite and axon growth, and synapse plasticity formation and plays a key role in neural circuit formation. At present, the Glu-CEST technique has been successfully applied in clinical trials concerning cerebral infarction and cranial trauma, providing a reference for this study (Lin et al., 2018; Mao et al., 2019). However, there are limited data investigating the value of Glu-CEST in neonates with acute bilirubin encephalopathy.

The aim of this study was to investigate the utility of combining ^1^H-MRS, Glu-CEST and conventional MRI in assessing the condition and prognosis of acute bilirubin encephalopathy patients and to understand the mechanism of nerve injury in this disease.

## Materials and methods

### Study participants

This study was conducted on 31 neonates with acute bilirubin encephalopathy at our hospital from September 2019 to February 2021. The inclusion criteria were (i) full-term infants with gestational age > 37 weeks, (ii) presence of clinical manifestations including yellow skin, elevated total bilirubin, an altered conscious state, abnormal muscle tone and reflexes, seizures, and poor feeding, which was consistent with neonatal bilirubin encephalopathy, or showing manifestations of acute bilirubin encephalopathy *via* brain MRI plain scan (Wang et al., 2008), (iii) complete clinical information. The exclusion criteria included presence of any other congenital neurologic disorders. Moreover, 16 healthy neonates were recruited during the same period as the normal control group. This study was approved by the Research Ethics Committee of our hospital. Informed consent was obtained from participants before the examination.

Based on bilirubin-induced neurologic dysfunction (BIND) scores including the condition of mental status, muscle tension and crying, 31 neonates with acute bilirubin encephalopathy were divided into the mild subgroup (BIND score ≤ 3, *n* = 21) and the moderate and severe subgroup (3 < BIND score ≤ 9, *n* = 10).

### MRI imaging

All neonates were underwent cranial conventional MRI, ^1^H-MRS and Glu-CEST evaluations after sedation with oral chloral hydrate 30 mg/kg. All scans were obtained with a 3.0-T scanner (Sigma; GE Healthcare, Milwaukee, WI, USA) with an 8-channel head coil.

Sequences of conventional MRI performed were as follows: axial and sagittal T1-weighted Fluid attenuated Inversion Recovery (T1W-FLAIR) imaging (TR: 1,750 ms, TE: 24 ms), axial T2-weighted Fast Spin Echo (T2W-FSE) imaging (TR: 4,600 ms, TE: 105 ms), and axial DWI (TR: 5,200 ms, TE: 75 ms) with slice thickness/spacing 4 mm/0.5 mm, slice number: 25, FOV 240 × 240 mm^2^.

**FIGURE 1:**
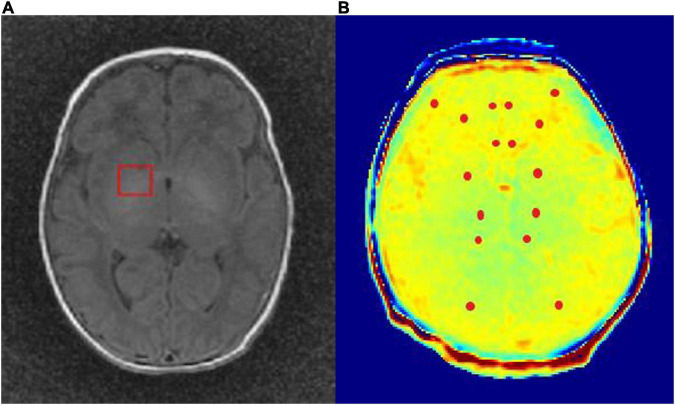
**(A)** For ^1^H-MRS acquisition, the level of the right basal ganglia was selected for the region of interest (ROI). **(B)** The ROI of glutamate chemical exchange saturation transfer (Glu-CEST) image post-processing included bilateral basal ganglia, thalamus, frontal cortex, frontal white matter, genu of the corpus callosum, cingulate gyrus, occipital white matter, and medial geniculate body.

For ^1^H-MRS acquisition, the level of the right basal ganglia was selected for the region of interest (ROI) (see Figure 1A). Sequence performed was single voxel point resolved spectroscopy sequence (TR = 1,500 ms, TE = 35 ms, voxel wide = 2 cm, length = 2 cm, thickness = 1 cm, acquisition time = 3 min 48 s). Six saturation bands were manually placed on the top, bottom, front, back, left, and right.

The Glu-CEST scan was based on a magnetization transfer (MT)–prepared gradient echo (GRE) MRI sequence with the following settings: TR = 50 ms, TE = 3.1 ms, FOV = 240 × 240 mm^2^, matrix = 128 × 128, NEX = 1, slice thickness = 5 mm, bandwidth = 15.63 kHz. MT saturation pulse was a Fermi pulse with a bandwidth of 8 ms and a flip angle of 600°. Forty-one equidistant frequency offsets from 5 to –5 ppm and S0 images were acquired.

### Data processing

^1^H-MRS processing: The raw P File of single-voxel ^1^H-MRS scanning was exported from the GE offline workstation and then imported to the LCModel software (LCModel Inc., Oakville, ON, Canada), followed by an analysis of the frequency spectrum of major metabolites. To improve data quality, results with standard deviations of less than 20% were selected. The absolute concentrations of metabolites were obtained by post-processing using LCModel software, which were the actual concentrations of the metabolites (mmol/g or mmol/L).

All CEST image processing were performed using MATLABR2013b (Mathworks, Natick, MA, USA). The Glu-CEST% value stood for the contrast variation of the Glu-CEST image, and the ROI included bilateral basal ganglia, thalamus, frontal cortex, frontal white matter, genu of the corpus callosum, cingulate gyrus, occipital white matter, and medial geniculate body (see Figure 1B). The mean Glu-CEST% value of every ROI was acquired, and each measurement was performed 3 times to reduce measurement error with the results averaged. The Glu-CEST contrast map was generated using the following equation (Mao et al., 2019):


(1) $$\text{GluCEST}=\frac{S\,\left(-3\,\text{ppm}\right)-S\,\left(+3\,\text{ppm}\right)}{S\,0}$$

### Follow-up

All neonates with acute bilirubin encephalopathy had return visits at our hospital 3 months after discharge and were assessed by rehabilitation physicians using the Gesell developmental schedules. Evaluations were conducted from fine movement, large movement, language ability, response to subject, and people. Developmental quotients (DQ) is an important indicator in the Gesell developmental schedules, and the DQ score stands for the growth rate of infants (76–85 points, normal; 55–75 points, mild developmental delay; 40–54 points, moderate developmental delay; 25–39 points, severe developmental delay; < 25 points, very severe developmental delay). Additionally, hearing screening was performed by otolaryngologists.

### Statistical analysis

All statistical data were analyzed using SPSS 22.0 software (IBM, Armonk, NY, USA). Continuous variables were expressed as mean ± standard deviation (SD) or median and interquartile range where appropriate. Continuous parameters were checked for the normality of distribution using the Shapiro–Wilk test and compared using the unpaired *t*-test or Mann-Whitney *U* test with normal and non-normal distributions, respectively. Differences among multiple groups were tested with one-way analysis of variance or Kruskal-Wallis *H* test followed by the Bonferroni *post hoc* analysis. The correlations between statistically significant indicators of imaging with peak serum TSB levels or DQ scores were evaluated using the Pearson’s correlation analysis or the Spearman rank correlation (r) coefficient, respectively. *p* < 0.05 was considered statistically significant.

## Results

### Characteristics of study participants

A total of 31 neonates with acute bilirubin encephalopathy were included in the analysis and were all full-term with 22 male infants and 9 female infants and serum total bilirubin (TSB) levels ranging from 299.5 to 818.4 μmol/L. The 31 cases were assigned to the mild subgroup (*n* = 21) and moderate and severe subgroup (*n* = 10) according to the BIND score. In addition, 16 healthy full-term neonates were randomly recruited during the same period with 10 male infants and 6 female infants. The baseline population characteristics are listed in Table 1. No significant difference was identified in gender, birth weight, and gestational age between the case group and the control group (*p* = 0.555, *p* = 0.668, *p* = 0.095, respectively).

**TABLE 1:** Baseline characteristics of study participants.

	Case group	Control group	*p*-value
*n*	31	16	…
Sex	22M/9F	10M/6F	0.555
Birth weight (kg)	3.32 ± 0.38	3.27 ± 0.31	0.668
Gestational age (d)	273 ± 8	277 ± 6	0.095

Data are expressed as mean ± SD or n of patients.

### Conventional MRI findings

*Via* conventional MRI scan, 13 out of 31 neonates with acute bilirubin encephalopathy manifested hyperintensity on T1WI in the globus pallidum region (8 from the mild subgroup, 8/21, 38.1%; 5 from the moderate and severe subgroup, 5/10, 50%), presenting a symmetrical outcome.

**FIGURE 2:**
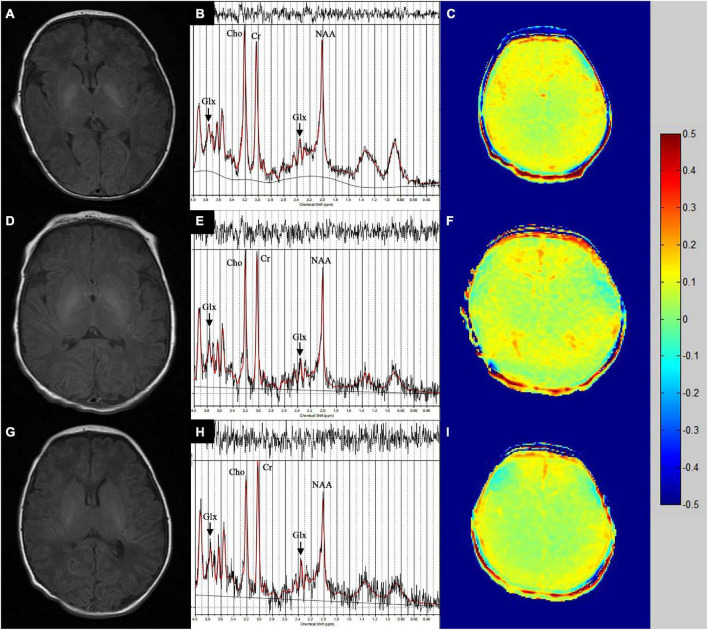
**(A)** A case with acute moderate and severe bilirubin encephalopathy (BIND score = 7) and **(D)** a case with acute mild bilirubin encephalopathy (BIND score = 3) manifested hyperintensity on T1WI in bilateral basal ganglia. The positive rate of bilateral basal ganglia T1WI hyperintensity increased along with rising disease severity. **(G)** No aberrant signal was observed in the bilateral basal ganglia of the healthy neonates. **(B,E,H)** ^1^H-MRS images of right basal ganglia in the moderate and severe subgroup, the mild subgroup and the control group. **(C,F,I)** Glu-CEST image post-processing in the moderate and severe subgroup, the mild subgroup and the control group.

The positive rate of the globus pallidum region T1WI hyperintensity increased along with rising disease severity (see Figures 2A, D). In addition, no aberrant signal was observed in the bilateral basal ganglia of the healthy neonates (see Figure 2G).

### ^1^H-MRS findings

^1^H-MRS images of right basal ganglia in the moderate and severe subgroup, the mild subgroup and the control group were presented in Figures 2B, E, H. Absolute concentration values of each metabolite in the right basal ganglia of the case group and the control group were summarized in Table 2. The case group had elevated Choline-containing Compounds (Cho) and γ-aminobutyric acid (GABA) absolute concentrations compared to the normal control group (*p* = 0.037, *p* = 0.038, respectively). No significant differences in the absolute concentrations of Creatine (Cr), Glutamate (Glu), MyoInositol (mI), N-Acetylaspartate (NAA), and microtubule-associated protein tau (Tau) were reported between two groups (*p* = 0.678, *p* = 0.574, *p* = 0.346, *p* = 0.171, *p* = 0.246, respectively).

**TABLE 2:** Absolute concentration values of each metabolite in the right basal ganglia of the case group and the control group.

	Control group	Case group	*p*-value
*n*	16	31	…
Cr (mmol/L)	2.958 ± 0.543	3.036 ± 0.794	0.678
Cho (mmol/L)	2.411 (2.306, 2.567)	2.587 (2.457, 2.710)	0.037
GABA (mmol/L)	0.757 ± 0.472	1.009 ± 0.369	0.038
Glu (mmol/L)	7.141 ± 1.184	6.942 ± 1.102	0.574
mI (mmol/L)	6.843 (6.265, 7.122)	6.785 (3.373, 7.370)	0.346
NAA (mmol/L)	4.440 ± 0.514	4.714 ± 0.649	0.171
Tau (mmol/L)	2.313 ± 0.829	2.581 ± 0.771	0.246

Data are expressed as mean ± SD, median (interquartile range), or n of patients. Cr indicates Creatine; Cho, Choline-containing Compounds; GABA, *γ*-aminobutyric acid; Glu, Glutamate; mI, MyoInositol; NAA, N-Acetylaspartate; Tau, microtubule-associated protein tau.

Absolute concentration values of each metabolite in the right basal ganglia of acute bilirubin encephalopathy in different severity groups were summarized in Table 3. Compared with the normal control group, the absolute concentration of GABA of the moderate and severe subgroup was significantly larger (*p* < 0.05). In addition, TSB level of the case group was positively associated with the absolute concentrations of GABA and Tau (*r* = 0.398, *p* = 0.006; *r* = 0.402, *p* = 0.005).

**TABLE 3:** Absolute concentration values of each metabolite in the right basal ganglia of acute bilirubin encephalopathy in different severity groups.

	Control group	Mild subgroup	Moderate and severe subgroup	*p*-value
*n*	16	21	10	…
Cr (mmol/L)	2.958 ± 0.543	3.161 ± 0.822	2.816 ± 0.679	0.416
Cho (mmol/L)	2.411 (2.306, 2.567)	2.535 (2.356, 2.701)	2.660 (2.523, 2.743)	0.064
GABA (mmol/L)	0.757 ± 0.472	0.953 ± 0.403	1.183 ± 0.270*	0.041
Glu (mmol/L)	7.141 ± 1.184	7.162 ± 1.187	6.496 ± 0.672	0.256
mI (mmol/L)	6.843 (6.265, 7.122)	6.687 (6.425, 7.390)	7.091 (6.183, 7.485)	0.638
NAA (mmol/L)	4.440 ± 0.514	4.666 ± 0.714	4.768 ± 0.494	0.361
Tau (mmol/L)	2.313 ± 0.829	2.405 ± 0.643	3.004 ± 0.872	0.069

Data are expressed as mean ± SD, median (interquartile range), or n of patients.

*p < 0.05 was considered to indicate a statistically significant difference between subgroup and control group.

### Glu-CEST findings

We screened all images collected, and those with artifacts were excluded. Glu-CEST image post-processing in the moderate and severe subgroup, the mild subgroup and the control group were presented in Figures 2C, F, I. The Glu-CEST% values of the case group (*n* = 22) and control group (*n* = 15) were analyzed in Table 4. Compared with the normal control group, the Glu-CEST% values in the left basal ganglia, right thalamus, left frontal cortex and bilateral medial geniculate body of the case group was significantly larger (*p* = 0.017, *p* = 0.043, *p* = 0.024, *p* = 0.019, *p* = 0.007, respectively). The Glu-CEST% values of acute bilirubin encephalopathy in different severity groups were summarized in Table 5. The moderate and severe subgroup had higher Glu-CEST% values in the left basal ganglia, right thalamus, and bilateral medial geniculate body than the normal control group (all *p* < 0.05). Compared with the mild subgroup, the Glu-CEST% values in the bilateral medial geniculate body of the moderate and severe subgroup were significantly larger (all *p* < 0.05). In addition, TSB level of the case group was positively associated with the Glu-CEST% values in the left basal ganglia, right thalamus, and bilateral medial geniculate body (*r* = 0.576, *p* < 0.001; *r* = 0.554, *p* = 0.001; *r* = 0.625, *p* < 0.001; *r* = 0.656, *p* < 0.001; respectively).

**TABLE 4:** Glutamate chemical exchange saturation transfer (Glu-CEST)% values for each ROI of the case group and the control group.

Position	Control group	Case group	*p*-value
*n*	15	22	…
LBG	2.776 ± 0.667	3.463 ± 1.001	0.017
RBG	3.583 ± 0.731	3.861 ± 0.937	0.342
LTH	2.240 (1.740, 2.728)	2.742 (2.130, 4.029)	0.051
RTH	2.416 ± 0.937	3.044 ± 0.860	0.043
LFC	1.726 ± 0.543	2.254 ± 0.823	0.024
RFC	1.679 (1.294, 2.165)	1.784 (1.467, 2.550)	0.567
LFWM	5.183 (4.803, 6.948)	5.905 (4.432, 7.471)	0.546
RFWM	5.536 (4.848, 6.880)	6.466 (4.429, 8.405)	0.578
LGCC	6.130 ± 1.540	5.753 ± 1.901	0.528
RGCC	5.972 ± 1.411	6.048 ± 1.899	0.895
LCG	6.712 ± 2.044	6.305 ± 2.659	0.621
RCG	7.589 (5.515, 7.976)	7.105 (4.819, 8.629)	0.951
LOWM	5.991 ± 1.927	5.926 ± 1.701	0.915
ROWM	6.031 (5.170, 7.620)	6.435 (4.803, 7.336)	0.867
LMGB	2.792 ± 1.328	4.452 ± 2.362	0.019
RMGB	2.821 ± 1.303	4.652 ± 2.215	0.007

Data are expressed as mean ± SD, median (interquartile range), or n of patients. LBG, left basal ganglia; RBG, right basal ganglia; LTH, left thalamus; RTH, right thalamus; LFC, left frontal cortex; RFC, right frontal cortex; LFWM, left frontal white matter; RFWM, right frontal white matter; LGCC, left genu of the corpus callosum; RGCC, right genu of the corpus callosum; LCG, left cingulate gyrus; RCG, right cingulate gyrus; LOWM, left occipital white matter; ROWM, right occipital white matter; LMGB, left medial geniculate body; and RMGB, right medial geniculate body.

**TABLE 5:** Glutamate chemical exchange saturation transfer (Glu-CEST)% values for each ROI of different severity groups.

Position	Control group	Mild subgroup	Moderate and severe subgroup	*p*-value
*n*	15	17	5	…
LBG	2.684 (2.491, 2.967)	3.046 (2.513, 3.810)	4.201 (3.856, 4.965)*	0.007
RBG	3.442 (3.181, 4.162)	3.815 (3.371, 4.038)	3.790 (3.363, 5.391)	0.495
LTH	2.240 (1.740, 2.728)	2.604 (2.081, 3.203)	4.032 (2.274, 4.578)	0.060
RTH	2.416 ± 0.937	2.873 ± 0.629	3.620 ± 1.326*	0.034
LFC	1.726 ± 0.543	2.262 ± 0.772	2.224 ± 1.081	0.114
RFC	1.679 (1.294, 2.165)	1.706 (1.383, 2.300)	2.643 (1.527, 4.041)	0.318
LFWM	5.183 (4.803, 6.948)	5.569 (4.380, 6.850)	6.874 (4.886, 9.760)	0.428
RFWM	5.536 (4.848, 6.880)	5.208 (4.413, 7.414)	8.650 (5.245, 13.528)	0.259
LGCC	6.130 ± 1.540	5.543 ± 1.555	6.469 ± 2.912	0.487
RGCC	5.972 ± 1.411	5.942 ± 2.049	6.407 ± 1.391	0.864
LCG	6.712 ± 2.044	6.085 ± 2.737	7.057 ± 2.497	0.655
RCG	7.589 (5.515, 7.976)	6.888 (4.695, 8.305)	8.081 (6.331, 9.182)	0.383
LOWM	5.991 ± 1.927	5.437 ± 1.481	7.589 ± 1.444	0.054
ROWM	6.074 ± 1.865	5.884 ± 1.697	7.955 ± 3.061	0.124
LMGB	2.792 ± 1.328	3.634 ± 1.585	7.238 ± 2.590*^&^	<0.001
RMGB	2.821 ± 1.303	3.788 ± 1.369	7.590 ± 2.097*^&^	<0.001

Data are expressed as mean ± SD, median (interquartile range), or n of patients. LBG, left basal ganglia; RBG, right basal ganglia; LTH, left thalamus; RTH, right thalamus; LFC, left frontal cortex; RFC, right frontal cortex; LFWM, left frontal white matter; RFWM, right frontal white matter; LGCC, left genu of the corpus callosum; RGCC, right genu of the corpus callosum; LCG, left cingulate gyrus; RCG, right cingulate gyrus; LOWM, left occipital white matter; ROWM, right occipital white matter; LMGB, left medial geniculate body; and RMGB, right medial geniculate body.

*p < 0.05 vs. control group.

^&^*p* < 0.05 vs. mild subgroup.

The correlation between the absolute concentration of glutamate (Glu) in the right basal ganglia of the case group (*n* = 22) and the Glu-CEST% value were evaluated. It was shown that the absolute concentration of Glu in the right basal ganglia was positively associated with the Glu-CEST% value (*r* = 0.483, *p* = 0.002).

### Gesell evaluation and hearing screening findings

A total of 10 neonates with acute bilirubin encephalopathy (6 from the moderate and severe group, 4 from the mild group) were followed up in this analysis for 3 months. 6 cases had DQ scores at 55–75 points with mild developmental delay, 1 case had DQ score at 40–54 points with moderate developmental delay, 3 cases had DQ scores at 25–39 points with severe developmental delay. Meanwhile, 3 cases showed hearing impairment and were diagnosed with auditory neuropathy after the clinical hearing screening, which was a sequela of acute bilirubin encephalopathy. A correlation analysis was performed between the imaging data acquired at the initial diagnosis and their DQ scores for the 10 cases, illustrating that the DQ scores was negatively related to the Glu-CEST% values in the left basal ganglia (*r* = −0.888, *p* = 0.008).

## Discussion

This research revealed that the case group had elevated Cho absolute concentrations compared to the normal control group. This was also similarly observed by Sarı et al. (2015). Previous study showed that Cho peak level significantly increases when neurons undergo necrosis and other pathological changes (Yang et al., 2016). This is consistent with our experimental results. Thus, we believe that neuron membrane metabolism disorder when bilirubin acts on immature neurons, which leads to the increase of Cho concentration. Additionally, compared with the normal control group, the absolute concentration of GABA of the case group was also significantly larger. GABA level is regulated by glutamate synthesis catalyzed by glutamate α-decarboxylase (GAD) and glutamate metabolism catalyzed by GABA transaminase (GABA-T) (Shungu et al., 2016). The synthesis and metabolism of GABA occur in neurons, and it also acts as an excitatory neurotransmitter in the developing nervous system. The major affected brain areas in acute bilirubin encephalopathy are areas containing γ-aminobutyric acid-ergic neurons, including globus pallidus, substantia nigra, hippocampus, cerebellum, and several auditory brainstem nuclei (i.e., ventral cochlear nuclei, and several superior olivary nuclei) (Shungu et al., 2016). Previous studies have shown that GABA neurons in neonatal rats develop earlier than glutamate neurons, so GABA neurons play a full and partial excitatory role in embryonic and early development (Ben-Ari, 2002). Previous study also showed that rats with bilirubin encephalopathy had significantly raised GABA in the hippocampus (Hu et al., 2014). Taken together, GABA and glutamate neurons are both excitatory neurons among developing immature neurons, which explains why the GABA absolute concentration increased in the basal ganglia of neonates from the moderate and severe subgroup and GABA level was positively related to TSB level. Moreover, GABA neuron excitability injures the developing neurons to some extent, presenting severer injuries under higher GABA concentrations and severer illness. Hence, we could infer that the increase in the absolute concentration of Cho and GABA metabolites in the right basal ganglia of neonates with acute bilirubin encephalopathy may be due to the increase of GABA concentration, resulting in excitatory damage to developing neuronal cells, which leads to the destruction of cell membrane integrity and further causes the increase of Cho concentration.

Glutamate chemical exchange saturation transfer (Glu-CEST) is a new noninvasive *in vivo* imaging method for oriented brain glutamate analysis and has markedly greater sensitivity and higher spatial resolution than the ^1^H-MRS method (Cai et al., 2012). In this study, brain areas with elevated Glu-CEST% values were basal ganglia, thalamus, frontal cortex, and medial geniculate body, and most of them are damage-prone regions in acute bilirubin encephalopathy. In immature astrocytes, extracellular Glu level was increased (Martínez-Contreras et al., 2002). It is well-known that increased glutamate release and overactivated glutamate receptors bring about excitotoxicity, astrogliosis, and neuron death (Mattson, 2003; Falcão et al., 2005), especially when cells are exposed to adverse conditions such as hypoxia, ischemia, increased oxidative stress, toxins, or other pathogens (Martínez-Contreras et al., 2002).

It was reported that bilirubin encephalopathy patients have a high incidence of sensorineural hearing loss and aberrant brainstem auditory evoked response, partially due to glutamatergic projections to the cochlear nucleus *via* auditory nerves and excitotoxic hearing loss induced by hyperbilirubinemia (Conlee and Shapiro, 1991). In this study, 3 cases who had severe bilirubin elevation on admission with BIND scores > 3 points showed hearing impairment during follow-ups. In addition, the moderate and severe subgroup had markedly increased Glu-CEST% values in the bilateral medial geniculate body versus the mild subgroup and the normal control group. Therefore, we can guess whether Glu-CEST% values can preliminarily evaluate the condition of auditory network impairment by bilirubin. According to the results of Gesell Developmental Schedules of the case group at the return visit 3 months later, DQ scores were negatively related to the Glu-CEST% values of the left basal ganglia, suggesting that greater glutamate accumulation in the basal ganglia represented poorer prognosis and lower brain development level in acute bilirubin encephalopathy.

In this experiment, the absolute metabolic concentration of glutamate treated by ^1^H-MRS was compared with the Glu-CEST% value of the corresponding sites, and found that there were obviously positive and statistically significant between ^1^H-MRS and Glu-CEST%. In this experiment, Glu-CEST technology can determine and analyze the glutamate value of multiple ROI at the same level, which can further evaluate the excitatory neurotoxicity of glutamate. ^1^H-MRS technology can quantitatively determine more metabolites. In the subsequent analysis of the correlation between DQ score and each index, the Glu-CEST% value in the left basal ganglia was significantly negatively correlated with the DQ score, which shows that Glu-CEST technology has a high sensitivity for the evaluation of prognosis in children with acute bilirubin encephalopathy. Therefore, ^1^H-MRS and Glu-CEST technology are complementary, and the combination of both can assess the metabolic level in the vulnerable areas of acute bilirubin encephalopathy in newborns, but from the level of glutamate metabolism, Glu-CEST technology is more sensitive than ^1^H-MRS technology.

There were some limitations to this study. First, the present study had a relatively limited study population, which was restricted to patients who fulfilled predefined inclusion criteria. Second, sample size obtained in follow-up was small. Further investigations are needed to clarify and understand the mechanism of nerve injury in acute bilirubin encephalopathy.

## Conclusion

This study demonstrated that the combination of ^1^H-MRS and Glu-CEST techniques can monitor the intracerebral metabolite level of acute bilirubin encephalopathy and evaluate the illness severity. It added the potential role for ^1^H-MRS and Glu-CEST techniques to assess nerve injury in neonatal acute bilirubin encephalopathy.

## Data availability statement

The original contributions presented in this study are included in the article/supplementary material, further inquiries can be directed to the corresponding author.

## Ethics statement

The studies involving human participants were reviewed and approved by the Research Ethics Committee of the Second Affiliated Hospital, Shantou University Medical College. Written informed consent to participate in this study was provided by the participants’ legal guardian/next of kin.

## Author contributions

QL: investigation, data curation, formal analysis, conceptualization, writing—original draft, and writing—review and editing. WZ: supervision, formal analysis, conceptualization, writing—original draft, writing—review and editing, project administration, and funding acquisition. LC: methodology, software, and formal analysis. HZ, HT, and GZ: investigation. All authors contributed to the article and approved the submitted version.
